# Divergence of imprinted genes during mammalian evolution

**DOI:** 10.1186/1471-2148-10-116

**Published:** 2010-04-29

**Authors:** Barbara Hutter, Matthias Bieg, Volkhard Helms, Martina Paulsen

**Affiliations:** 1 Lehrstuhl für Computational Biology, Universität des Saarlandes, Postfach 151150, D-66041 Saarbrücken, Germany; 2 Lehrstuhl für Genetik/Epigenetik, Universität des Saarlandes, Postfach 151150, D-66041 Saarbrücken, Germany

## Abstract

**Background:**

In contrast to the majority of mammalian genes, imprinted genes are monoallelically expressed with the choice of the active allele depending on its parental origin. Due to their special inheritance patterns, maternally and paternally expressed genes might be under different evolutionary pressure. Here, we aimed at assessing the evolutionary history of imprinted genes.

**Results:**

In this study, we investigated the conservation of imprinted genes in vertebrate genomes and their exposition to natural selection. In a genome-wide comparison, orthologs of imprinted genes show a stronger divergence on cDNA and protein level in mammals. This pattern is most pronounced for maternally expressed genes in rodents in comparison to their non-rodent orthologs. The divergence is not attributable to increased mutation of CpG positions. It is contrasted by strong conservation of paternally expressed genes in mouse and rat. Interestingly, we found that the early divergence of imprinted genes was accompanied by an unusually strict conservation of their paralogs.

**Conclusions:**

The apparent degeneration of maternally expressed genes may reflect a relaxation of selective pressure due to counteracting effects on maternal and embryonic fitness. Functional redundancy provided by the presence of highly conserved (non-imprinted) paralogs may have facilitated the divergence. Moreover, intensification of imprinting in modern rodents seems to have shifted the evolutionary fate of imprinted genes towards strong purifying selection.

## Background

Deciphering the evolution of eukaryotic genes is a key to understanding their function in different species. As nearly all mammals are diploid organisms, most of their genes are expressed from both parental alleles. Nevertheless, a number of genes do not obey this rule, notably so-called imprinted genes. They acquire specific epigenetic marks in the parental germ lines which are the cause of monoallelic expression after fertilization. Consequently, it depends on the parental origin which allele of an imprinted gene gets inactivated. Genomic imprinting has been observed in the endosperm of flowering plants and in the animal kingdom. In the latter, imprinted genes have so far only been discovered in therian species [1,2]. Monoallelic expression may thus be evolutionary advantageous for particular functions. Based on the finding that genomic imprinting is a specific feature of species in which embryo and mother are in direct contact to each other, imprinted genes were hypothesized to regulate maternal nutrient supply during embryonic development. The kinship theory names the different interests of the parents in the wellbeing of the embryo as a possible evolutionary origin of imprinting [3]. According to this model, the mother aims at saving resources for subsequent pregnancies that are possibly fathered by different males, whereas the father is interested in the maximal exploitation of maternal resources in favor of his own offspring. This would result in a selective pressure towards silencing of growth promoting genes in the female germ line, and of growth suppressing genes in the male germ line. In both cases the result would be monoallelic expression of the respective genes after fertilization.

Due to their monoallelic silencing, imprinted genes might react differently on natural selection than biallelically expressed genes. In fact, their functional haploidy is reminiscent of the scenario for genes on the X chromosome, on which both positive and negative selection act more efficiently than on the autosomes (reviewed in [4]). Deleterious mutations affecting the expressed allele will be subject to selective elimination whereas beneficial ones would provide immediate advantages. On the other hand, the inactive allele may accumulate mutations that remain unexposed as long as the sex of the transmitting parent does not switch [5]. Imprinted genes have been supposed to play key roles in the mammalian embryo and placenta. Hence, evolution of imprinting and speciation of placental mammals might be linked. Evolution of imprinting might have subjected the affected genes to strong purifying selection, or might have triggered a strong positive selection towards species-specific functions. Fitting into the second scenario it has been reported that the evolution of imprinting of the *MEDEA *gene in *Arabidopsis *species might have been initiated by a duplication event and that the evolution of the imprinted duplicate was accompanied by strong positive selection [6]. Interestingly, also the evolution of mammalian imprinted genes was suggested to be influenced or even triggered by duplication events [7,8].

Studies on a limited number of imprinted genes in mouse and rat did not provide evidence for conspicuous mutation rates or positive selection in the rodent lineage [9,10]. Due to their functions as growth regulators, special attention has been paid to the imprinted genes *Igf2 *and *Igf2r*. Whereas DNA sequences that encode the interface region of the IGF2R and IGF2 proteins are highly conserved, the signal sequence of IGF2R that determines the protein's location in the cell is strikingly divergent between mouse and rat as well as between human and cow [9,10]. Indication of positive selection on IGF2 in viviparous fish species that developed placenta-like structures suggests that evolutionary adaptations of growth factor encoding genes might predate their imprinting [11].

To date, about 90 imprinted genes have been identified in human and mouse [12,13]. In order to assess their evolutionary history in placental mammals, we analyzed sequence conservation of maternally and paternally expressed genes and their paralogs in a genome-wide comparison. We show that the evolution of imprinted genes is characterized by an accelerated divergence at DNA and protein level in rodent ancestors. Moreover, the existence of paralogous genes seems to have facilitated divergence of imprinted genes.

## Results

### Maternally expressed genes show reduced conservation

In order to assess the evolutionary conservation of imprinted genes, we selected 58 pairs of orthologous protein encoding human and mouse genes for which imprinting has been reported in at least one of the two species in the Otago Catalogue of Imprinted Genes [12,13] and in the literature (see additional file 1). These imprinted genes were compared on a genome-wide scale to all genes annotated in the HomoloGene database [14]. This database provides information derived from alignments of orthologous RefSeq cDNA and protein sequences. We extracted identity values for the alignments of human cDNA and amino acid sequences, respectively, to their orthologs of mouse, rat, chimpanzee, dog, cow, and chicken.

Compared to genome-wide data, human and mouse ortholog pairs of maternally expressed genes are less conserved in terms of cDNA identity (p < 0.05) and the encoded proteins show a trend towards reduced identity (p < 0.06; Table 1, data for individual imprinted genes are given in additional file 1). Additionally, a prominent feature was the increased number of gaps - corresponding to insertions or deletions of amino acids - in the sequence alignments of proteins encoded by maternally expressed genes (p < 0.05). This might hint at an increase of exon insertions or deletions, respectively.

**Table 1:** HomoloGene data for human-mouse orthologous gene pairs

group	genes	protein identity ± std.dev. (%)	cDNA identity ± std.dev. (%)	Ka/Ks ± std.dev.	Ks ± std.dev.
imprinted	53	83.7 ± 11.3	83.4 ± 6.4	0.148 ± 0.113	0.655 ± 0.232

maternally expressed	26	82.5 ± 10.1*	82.5 ± 5.9**	0.161 ± 0.116*	0.674 ± 0.179

paternally expressed	27	84.8 ± 12.4	84.3 ± 6.9	0.136 ± 0.110	0.639 ± 0.272

genome	16,582^a^	85.6 ± 11.7	84.4 ± 6.5	0.129 ± 0.109	0.642 ± 0.228

^a ^Ka/Ks rates are undetermined for 21 genes. * p < 0.1, ** p < 0.05 (Wilcoxon test for comparison of the respective group to genome data)

Comparing the human to dog, cow, and chimpanzee, maternally expressed genes exhibit the lowest identities on DNA and protein levels, thereby supporting an increased divergence of maternally expressed genes (additional file 2). However, the reduced conservation between human and non-rodent species is not statistically significant (p > 0.1). Paternally expressed genes have a similar level of sequence conservation as the genomic background in all pairwise comparisons of the human to other mammalian species.

As imprinted genes are associated with particular, allele-specific DNA methylation patterns, the observed divergence of their protein-encoding sequences might be due to an increased rate of CpG to TpG transitions. Addressing this issue we investigated the frequencies of silent CpG to TpG transitions at positions in the cDNA where these mutations would not change the encoded amino acid [10,15]. We found that both maternally expressed genes and the whole imprinted group exhibit insignificantly lower levels of silent CpG-TpG mismatches in the cDNA alignments compared to genome-wide data (p > 0.3). Hence, the increased divergence is not caused by increased deamination of methylated CpGs.

### Divergence of imprinted genes between rodents and other mammals

The low sequence identity of maternally expressed orthologs results apparently from an accelerated divergence in mouse or rodents. Further evidence for the latter hypothesis is provided by comparison of human and rat genes. The obtained data are consistent with those from mouse (additional file 3). Here, the reduced conservation of maternally expressed genes is even more significant (p < 0.04), which can be attributed to a higher number of substitutions in the rat [16]. Also between mouse and cow the conservation of all imprinted and the maternally expressed genes is lower than that of non-imprinted genes on the protein and cDNA levels (additional file 2). Comparisons with other species, for which fewer sequences of imprinted genes are available, show a tendency towards increased divergence of murine imprinted genes as well (additional file 2). Interestingly, comparing mammalian genes to their orthologs in chicken, a species without imprinting effects, did not reveal any changes in the conservation of imprinted genes that might be associated with the evolution of imprinting in early mammals.

Increased divergence of maternally expressed genes might have its cause in increased mutation rates, reduced purifying selection, or positive selection. Increased mutation rates are indicated by an elevated rate of synonymous substitutions (Ks) [9,10]. For human-mouse (Table 1) and mouse-cow (additional file 2) gene pairs, Ks rates are essentially similar in all groups, thereby contradicting a major influence of mutation rates. A commonly used method for estimating selection is determining the ratio of synonymous substitutions (Ks) and non-synonymous substitutions (Ka) per site in pairwise alignments of coding DNA [17]. In general, Ka/Ks ratios of below 0.25 indicate purifying selection [16] whereas Ka/Ks values larger than 1 are indicative of positive selection.

The genome-wide median of the Ka/Ks ratio is 0.100 for mouse-human gene pairs. Ka/Ks tends to be elevated for the group of 26 maternally expressed genes (median 0.124; p < 0.08) but this is not the case for the 27 paternally expressed ones (median 0.110; p > 0.7). The imprinted genes with the most elevated Ka/Ks values are the maternally expressed genes *Cdkn1c *(Ka/Ks = 0.465) and *Phlda2 *(Ka/Ks = 0.390), and the paternally expressed *Usp29 *(Ka/Ks = 0.384). As indicated by very low Ka/Ks values, the strongest purifying selection appears to act on the paternally expressed genes *Snrpn*, *Mest*, *Wt1*, and *Copg2 *(see also additional file 1). The Ka/Ks ratios of the 38 imprinted orthologs available for mouse and cow are tentatively elevated as well (additional file 2). In summary, the set of imprinted genes studied here does not contain genes with exceptionally high Ka/Ks ratios that indicate recent positive selection.

### Strong conservation of imprinted genes in modern rodents

Next, we wanted to know whether the reduced conservation of imprinted genes is due to divergence in a common ancestor of mouse and rat, or whether this evolutionary process is still ongoing in modern rodents. Intriguingly, comparison of mouse and rat orthologs (Table 2) shows that the conservation levels of imprinted genes are similar to those of all genes on the protein level (p > 0.6), and even higher on DNA level (p < 0.02). The latter fact is mostly caused by the paternally expressed genes whereas maternally expressed genes are similarly conserved as the genomic background. The Ka/Ks ratio of imprinted genes (median 0.12) is not significantly higher than genome-wide (median 0.10). Murine imprinted genes contain a median of 2.4 single nucleotide polymorphisms (SNPs) per 1 kb of coding sequence whereas the genome-wide median is 3.6 (p < 0.04). This depletion, which is not seen for human imprinted genes (medians 3.9 and 3.2, respectively; p > 0.4) strongly argues for ongoing purifying selection on the protein-coding regions of murine imprinted genes.

**Table 2:** HomoloGene data for mouse-rat orthologous gene pairs

group	genes	protein identity ± std.dev. (%)	cDNA identity ± std.dev. (%)	Ka/Ks ± std.dev.	Ks ± std.dev.
imprinted	46	94.9 ± 3.3	94.4 ± 2.1	0.137 ± 0.091	0.186 ± 0.074***

maternally expressed	26	94.5 ± 3.4	94.1 ± 2.0	0.147 ± 0.092	0.192 ± 0.069**

paternally expressed	20	95.5 ± 3.1	94.8 ± 2.3**	0.124 ± 0.091	0.178 ± 0.081**

genome	16,800^a^	93.2 ± 7.1	92.9 ± 4.0	0.147 ± 0.149	0.229 ± 0.108

^a^Ka/Ks rates are undetermined for 49 genes. ** p < 0.05, *** p < 0.001 (Wilcoxon test for comparison of the respective group to genome data)

Contrary to the pattern observed for mouse-human gene pairs, mouse-rat imprinted orthologs have a decreased rate of synonymous substitutions (p < 0.008) whereas the rate of nonsynonymous changes is not significantly elevated (p > 0.15). This finding holds for both paternally and maternally expressed genes and is in agreement with the results of Smith and Hurst [10]. Reduced Ks rates may hint at selection on silent sites related to alternative splicing and RNA secondary structure requirements [18]. Alternatively, a special chromatin structure of imprinted genes might reduce overall mutation rates in the germ lines, thereby leading to the observed lower synonymous substitution rates. A similar connection is not seen for human-mouse or human-chimpanzee orthologs (additional file 2), which might point at a rodent-specific evolutionary pattern.

### Reconstructing patterns of ancient evolution

**Figure 1:**
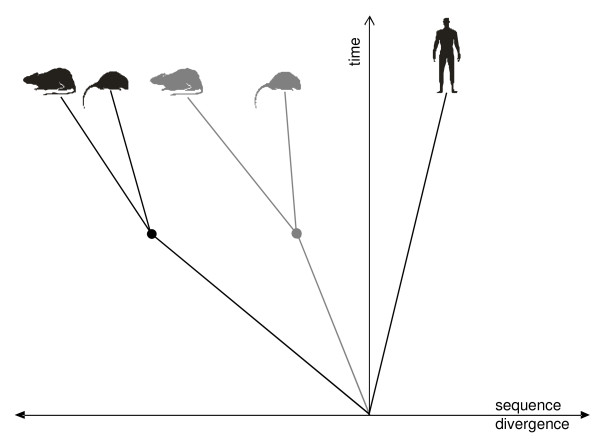
**Different patterns of divergence for imprinted and biallelically expressed genes**. Imprinted genes (black) supposedly evolved faster than biallelically expressed genes (gray) in the common ancestors of rodents. After the split of rat and mouse (dots), imprinted genes seem to have been subject to stricter purifying selection than biallelically expressed genes. The present conservation pattern reflects a high divergence of imprinted human-rodent orthologs as opposed to high conservation between mouse and rat.

The elevated conservation of imprinted genes between the two rodent species is in contrast to their divergence from their orthologs in other mammals. Hence, most of the discerning DNA changes must have taken place before the split of rat and mouse. Afterwards, imprinted genes were possibly subjected to strict purifying selection in mouse and rat (Figure 1). Since both mechanisms counteract and thus obscure the pattern in the sequences of extant species, we aimed at estimating conservation and evolutionary rates before the split of mouse and rat. From pairwise alignment data on human-mouse (*hs_mm*), human-rat (*hs_rn*) and mouse-rat (*mm_rn*), we estimated protein and cDNA identity as well as Ka and Ks rates between human and a common ancestor of mouse and rat (*hs_rodent*) using formula (1) for protein or cDNA identity given in percent and formula (2) for Ka or Ks rates.(1)

Formula (1) can be applied for sequences where *mm_rn *>*hs_mm *and *mm_rn *>*hs_rn*, which applies to the vast majority of all genes that are present for all three species in HomoloGene. In fact, we detected only 21 genes where the identity of alignments between human and rodent proteins was higher than that between mouse and rat. This can be due to differences in the alignments caused by events such as changes in exon usage during murine evolution. Likewise, formula (2) is not applicable for 40 cases in which the Ka or Ks rates are higher between the two rodent species than between human and rodent sequences.

With regard to the reconstructed rodent ancestor, sequence identities become higher but more discriminative between imprinted and all genes. Comparisons of the obtained values of 46 imprinted genes to 14,517 genome-wide reconstructions gave results consistent with the observations described above. Increased divergence of DNA and protein sequences (p < 0.03) and elevation of Ka (p < 0.03) of maternally expressed genes became slightly more significant. In addition we observed trends toward higher Ka values in the whole imprinted set (p < 0.06), and higher Ks and Ka/Ks rates of maternally expressed genes (p < 0.09).

An alternative option to trace different evolutionary constraints in the mammalian lineages is provided by the *PAML *package [19]. We constructed branch models including the cow as fourth mammalian species. In the imprinted group, the lineage leading to the rodent ancestor has similar Ka/Ks ratios as other lineages for 23 out of 34 genes. For the remaining eleven genes, the two-ratios model assuming a different Ka/Ks ratio is significantly more likely than the one-ratio model assuming the same Ka/Ks ratio for all branches (p < 0.05). For four of these genes (*Cdkn1c*, *Igf2r*, *Magel2*, and *Ndn*), the Ka/Ks ratio calculated by the two-ratios model is elevated in the lineage leading to the rodent ancestor. All four genes were found to have Ka/Ks rates above the median values for human-mouse (additional file 1) indicating that these genes - interestingly members of prominent imprinted regions - have been subject to species-specific shifts in evolutionary patterns. Since the PAML estimations of Ka/Ks are always lower than 1 for all lineages, the imprinted genes studied here apparently did not experience strong positive selection but rather relaxation of purifying constraints.

### Interacting evolution of imprinted genes and their paralogs

Identification of general evolutionary principles on the basis of simple sequence features crucially depends on the careful construction of suitable control groups. Having originated from the same ancestral gene, paralogs are supposed to fulfill similar (yet not identical) functions [20]. Therefore, comparing imprinted genes to their paralogs seemed an attractive opportunity to reveal differences in evolution that are not related to the biochemical functions of these genes *per se *but rather to their particular, monoallelic expression pattern. First, we analyzed whether there is an enrichment of paralogs in the imprinted gene group according to the Ensembl release 52 [21] annotations for 19,950 human autosomal protein-coding genes. To avoid a bias of genes with many paralogs, we chose for each gene the one that is listed first as its representative paralog. This paralog is the evolutionary most recent and in most cases also the one with the highest identity. For some imprinted genes (*Dlk1*, *Inpp5f, MAGEL2*, *NAP1L5*, *NDN*, and *Peg10*) the approach used here gave different results than previously reported in the literature [7,8,22,23] (additional file 1). This includes duplicates that were reported to originate from retrotransposition events from the X chromosome [8]. Nevertheless, 60.71% of the genes in the imprinted group possess a paralog, a slightly higher percentage than for all genes on human autosomes (48.22%, χ^2 ^test, p < 0.1). Interestingly, most duplication events predate the origin of mammals, also in the imprinted group. With the exception of *Ins1 *and *Ins2*, the imprinted genes studied here were not involved in duplication events after the split of the human and rodent lineages.

Several imprinted genes have been linked to paralogs on the X chromosome [7,8,24] and it has been speculated that imprinting and X chromosome inactivation may have co-evolved since they require similar mechanisms of gene regulation [8,25-27]. We found that the first paralogs of three imprinted genes (*DCN*, *HTR2A, USP29*) are located on the X chromosome. Additional three (*L3MBTL*, *SLC38A4*, *UBE3A*) have an X-linked paralog that is not the highest scoring one. The resulting 10.71% is no significant enrichment compared to the autosomal ratio of 5.19% (Table 3, χ^2 ^test, p > 0.1). Also in the mouse there is no significant enrichment of X-linked paralogs of imprinted genes.

**Table 3:** Pairs of genes and their paralogs

group	genes	with paralog	median number of paralogs	most recent paralog on X	has a paralog on X	average protein identity ± std.dev. (%)^a^
human imprinted	56	34*	2	3	6	47.12 ± 15.24**

human autosomes	19,950	9619	2	288	1035	56.85 ± 22.73

mouse imprinted	54	33*	2*	2	4	44.94 ± 18.02***

mouse autosomes	21,871	10919	3	309	1029	60.68 ± 24.07

^a^In cases where more than one paralog was available, protein identities were analyzed only for the gene and its evolutionary most recent paralog. * p < 0.1, ** p < 0.05, *** p < 0.001 (χ^2 ^or Wilcoxon test for comparison of the respective imprinted group to autosomal data)

Compared to genome-wide data, human imprinted genes show less identity with their most recent paralogs on protein level (p < 0.06, Table 3). In the mouse this relaxation in paralog conservation is more pronounced (p < 0.007), and is probably caused by the stronger divergence of imprinted genes in the rodent ancestor as described above. Taking the average identity of all paralogs per gene as a measure yields lower identity values without affecting the significance of the differences.

To investigate whether the existence of paralogs might influence the evolution of protein-coding imprinted genes, we investigated the conservation of gene orthologs in presence or absence of paralogs. In the entire genome and also in the case of imprinted genes, orthologs that possess paralogs are significantly more conserved between human and mouse, or mouse and rat than those without a paralog and have lower Ka/Ks ratios (p < 0.001, Table 4). Comparing imprinted genes with or without paralogs, respectively, to the corresponding groups of autosomal genes, reveals that imprinted genes with paralogs are subject to decreased conservation between human and rodents (p < 0.04) and tend towards a higher Ka/Ks ratio (p < 0.09) whereas there is no significant difference between genes without paralogs in both groups (p > 0.3). Between mouse and rat, genes with paralogs behave similarly in both groups (p > 0.2) but imprinted ones without paralogs show increased conservation on DNA level and a lower Ks ratio (p < 0.002). In all comparisons maternally and paternally expressed genes behaved similarly. Overall, genes with high numbers of paralogs did not behave differently from genes with only one or few paralogs.

**Table 4:** HomoloGene data for genes with or without paralogs

group	genes^a^	protein identity ± std.dev. (%)	cDNA identity ± std.dev. (%)	Ka/Ks ± std.dev.	Ks ± std.dev.
imprinted human-mouse with paralogs	32	85.0 ± 10.8**	83.6 ± 5.9**	0.131 ± 0.106*	0.678 ± 0.189

imprinted human-mouse without paralogs	20	82.7 ± 11.6	83.3 ± 7.3	0.165 ± 0.116	0.623 ± 0.294

genome human-mouse with paralogs	7235/ 7228	88.2 ± 10.5	85.7 ± 5.9	0.105 ± 0.096	0.625 ± 0.229

genome human-mouse without paralogs	7765/ 7756	83.7 ± 11.9	83.4 ± 6.4	0.145 ± 0.112	0.656 ± 0.212

imprinted mouse-rat with paralogs	28	94.8 ± 3.4	93.9 ± 2.0	0.120 ± 0.081	0.211 ± 0.066

imprinted mouse-rat without paralogs	18/ 17	95.1 ± 3.1	95.2 ± 2.2***	0.166 ± 0.102	0.148 ± 0.072***

genome mouse-rat with paralogs	7638/ 7636	94.3 ± 6.4	93.5 ± 3.4	0.125 ± 0.138	0.224 ± 0.096

genome mouse-rat without paralogs	7509/ 7505	93.0 ± 6.4	92.9 ± 3.4	0.155 ± 0.143	0.229 ± 0.082

^a^The second number refers to sequences available in the HomoloGene database for Ka/Ks analyses. * p < 0.1, ** p < 0.05, *** p < 0.002 (Wilcoxon test for comparison of the respective imprinted group to genome data)

Interestingly, the paralogs show a higher conservation than their imprinted counterparts and all genes in HomoloGene and remarkably lower Ka/Ks ratios between human-mouse, human-rat, and mouse-rat (Table 5). No differences were found between maternally and paternally expressed genes. Purifying selection thus seems to act far more strictly on the paralogs of rodent imprinted genes than on the imprinted genes themselves.

**Table 5:** HomoloGene data for paralogs of imprinted genes

species	genes	protein identity ± std.dev. (%)	cDNA identity ± std.dev. (%)	Ka/Ks ± std.dev.	Ks ± std.dev.
human-mouse	32	90.0 ± 10.4**	86.9 ± 6.0***	0.094 ± 0.092*	0.570 ± 0.185

human-rat	28	89.9 ± 11.0**	86.6 ± 6.3**	0.089 ± 0.083**	0.603 ± 0.193

mouse-rat	28	97.1 ± 2.2***	94.8 ± 1.7***	0.065 ± 0.042***	0.206 ± 0.065

* p < 0.1, ** p < 0.05, *** p < 0.01 (Wilcoxon test for comparison of the respective group to genome data). Data refer to the evolutionary most recent paralog of each imprinted gene.

## Discussion

### Divergence of imprinted genes in early eutherian evolution

Detailed genome-wide analyses on cDNA and protein level revealed an intriguing evolutionary pattern of imprinted genes. Compared to their (non-imprinted) chicken orthologs, imprinted genes show similar sequence identities and Ka/Ks distributions as all genes in the HomoloGene database. This suggests that in early mammals there was no specific evolutionary pressure on the protein-coding sequences of imprinted genes. In contrast, imprinted genes of mouse and rat show an elevated divergence of coding DNA sequences and proteins in comparison to their orthologs in other mammalian species, especially in the human, suggesting that there has been an increased divergence of imprinted genes in a common ancestor of mouse and rat. Although the reduced conservation was only significant for the rodent lineage, we cannot exclude that imprinted genes underwent a relaxation of selective constraints also in other species. The observed differences might be more pronounced in rodents due to their shorter generation times and effects of their larger population sizes [16]. One also has to keep in mind that the analysis of genes in species such as dog, cow, and chicken that have not been studied as long as human and mouse is hampered by a still low number and quality of available sequences.

In extant rodents, evolutionary processes seem to have shifted towards purifying selection. At present, murine imprinted genes are apparently under strong purifying selection as suggested by the reduction of SNPs. This pattern of evolution, i.e. an initial divergence followed by fixation, is believed to be typical for the evolution of new functions, for example of duplicated gene copies [28-30] or for the evolution of new species. Remarkably, the increased conservation in modern rodents coincides with a stricter conservation of typical DNA elements in imprinted genes in the mouse than in the human, such as intronic CpG islands [31].

### Antagonistic feedback effects of maternally expressed genes

Interestingly, the increased divergence of imprinted genes affects mostly maternally expressed genes. Especially for rodents, the reduction of embryonic growth by genes expressed from the maternal alleles may have provided evolutionary advantages along with changes in placentation [32,33]. Indeed, the maternally expressed genes *Cdkn1c *and *Phlda2 *that fulfill important functions in the mouse placenta have the highest Ka/Ks ratios of all imprinted genes. Thus, a possible explanation for the low conservation of maternally expressed genes might be the evolution of new functions in different lineages. However, the rather modest elevation of the Ka/Ks ratios argues rather for relaxed constraints than for positive selection.

An alternative scenario is the degeneration of (maternally expressed) growth repressors. These are likely to have different, counteracting effects on maternal and embryonic fitness: On the one hand a growth repressor might reduce the fitness of the embryo, on the other hand small embryos might increase the number of offspring. The counteracting effects on embryonic and maternal fitness should result in a reduced selective pressure on maternally expressed growth regulators, resulting in the observed relaxed conservation among mammalian species. Since there is no physical interaction between the embryo and the father, a similar effect of paternally expressed genes on paternal fitness is hardly conceivable. In support of this we found that the set of paternally expressed genes shows a similar or even higher level of conservation as the genome-wide background.

### Paralogs may facilitate divergence of rodent imprinted genes

We did not find evidence that gene duplication events originating on the X chromosomes are a key factor in the evolution of imprinted genes as it has been suggested previously [8]. However, we cannot exclude that the duplication of a few genes on the X chromosome and their translocation to the autosomes might have initiated the evolution of imprinted genomic regions. Elements that initially regulated only imprinting of the duplicated genes may have strengthened and started to influence also the expression of neighboring genes. Interestingly, the six genes in our study that possess X-chromosomal paralogs are distributed over different imprinting domains, thus representing potential originators.

**Figure 2:**
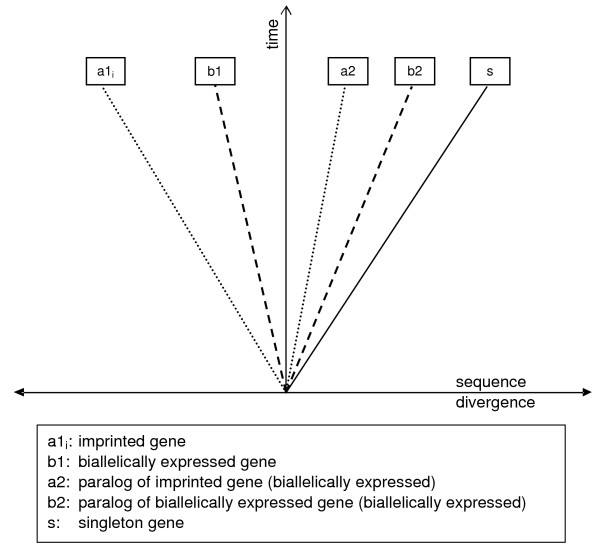
**Complementary divergence**. The orthologs of single copy genes (s) are more diverged than the orthologs of genes that possess paralogs. Regarding paralogous pairs of biallelically expressed genes (b1, b2), one is usually more diverged than the other. If an imprinted gene (a1_i_) has a paralog (a2), the imprinted gene itself is in most cases the more divergent one.

Although duplication events might not be directly linked to evolution of imprinted gene regulation, the existence of paralogs may have enabled a greater divergence of imprinted genes by relaxation of purifying selection. Genes that possess paralogs show evolutionary patterns that differ from those of singleton genes [28-30,34]. Notably, there is complementary divergence between pairs of paralogs, where imprinted genes seem to be concentrated in the group of the faster evolving duplicates (Figure 2). The strong conservation of their non-imprinted paralogs suggests that these genes may have compensated for the divergence by maintaining the original functions of the ancestral genes.

## Conclusions

The evolutionary fate of maternally expressed genes appears to be influenced by antagonistic effects on maternal and embryonic fitness, resulting in a relaxation of selective pressure compared to biallelically expressed genes. The observed divergence of imprinted genes might have been compensated by a restrictive conservation of their paralogs. In most cases the duplication events predated the split of the fish and mammalian lineages and thus the evolution of imprinting. Long-lasting functional redundancy may have allowed genes that later became imprinted to specialize in functions related to placental and embryonic development. Apparent purifying selection on paternally expressed genes in modern rodents might be linked with the intensification of imprinting in species with a pronounced conflict over maternal resources.

## Methods

### Gene selection

From the Otago Catalogue of Imprinted Genes [12,13] and the literature we selected 58 orthologous pairs of protein-coding genes in human and mouse. For these pairs, imprinting effects had been observed at least in one of the two species and the gene sequences could be localized with the UCSC Genome Browser [35] for human (March 2006 assembly hg18, NCBI build 36.1) and mouse (February 2006 assembly mm8, NCBI build 36.1). The genes are listed according to their parental expression in additional file 1.

### Analysis of orthologous and paralogous sequences

Genome-wide data on orthologous sequences were retrieved from HomoloGene release 62 [14]. We analyzed identities of nucleotide and protein sequences, rates of synonymous substitutions (Ks), rates of nonsynonymous substitutions (Ka), and their ratios for pairwise sequence alignments. In case there was more than one homolog per species, we chose the one with the highest protein sequence identity. Ka and Ks rates given in the database are calculated using the method of Nei and Gojobori [36]. Entries with Ks reported as -1 were discarded from Ks and Ka/Ks analyses.

Information on human and mouse paralogs was taken from Ensembl Release 52 [21] with the BioMart tool. Paralogs in this database are annotated for the longest transcript of a gene and sorted by taxonomy level: The first paralog is the evolutionary most recent one. Usually it corresponds to the best one in terms of identity of both the query and the target sequence. Thus we chose the first paralog listed for further analyses. The given sequences identities refer to the protein sequences.

### Alignment generation and identification of SNPs

For 12,143 genes, orthologous sequences of human, mouse, rat, and cow could be obtained via their identifiers in HomoloGene using the Entrez Programming Utilities [37]. We inferred the cDNAs of their longest open reading frames and aligned them with *transAlign *[38]. This program translates the cDNAs into the corresponding amino acid sequences and generates an alignment using ClustalW [39,40]. The resulting protein alignment is back-translated into a DNA alignment. Thus, frameshifts due to gaps that are not multiples of three are avoided. Silent CpG mutability was assessed by calculating the ratio of CpG-CpG pairs and CpG-TpG pairs with C at the third codon position in pairwise alignments of human-mouse cDNAs [10,15]. Single nucleotide exchanges and indels based on dbSNP version 129 [41] were assigned to the coding exons of all RefSeq genes using a local installation of the University of California Genome Browser and the associated tool kit [35] and analyzed with custom Perl scripts.

### Phylogenetic analysis

We used the program *codeml *from the *PAML *package [19] to construct branch models for each four-species alignment using the unrooted tree (human,(mouse, rat) #1, cow). The one-ratio model assigns the same Ka/Ks ratio to each branch, the alternative two-ratios model estimates a different ratio for the rodent ancestor branch marked by #1. Ks and Ka rates are calculated separately by *codeml *to fulfill the respective Ka/Ks. Genes with Ks = 0 and Ks > 2.5, which is a result of saturation, were omitted from further analyses because these data are unreliable. According to a χ^2 ^distribution with one degree of freedom, the two-ratios model provides a better fit than the one-ratio model on a significance level of p < 0.05 if twice the difference of the two reported log likelihood ratios is at least 2.71.

## Authors' contributions

BH organized the study, acquired the data, performed the statistical analyses, and drafted the manuscript. MB processed the HomoloGene and SNP data. VH participated in the interpretation of the data and revised the manuscript. MP initiated the study, contributed to its design and to the interpretation, and edited the manuscript. All authors read and approved the final manuscript.

## Supplementary Material

Additional file 1 **Conservation and existence of paralogs of protein-coding imprinted genes**. This Excel spreadsheet gives information about the conservation of the human-mouse imprinted genes used in this study and their paralogs. Click here for file

Additional file 2 **HomoloGene data for additional orthologous gene pairs**. This pdf file contains HomoloGene data for orthologous gene pairs of human-chimpanzee, human-cow, human-dog, human-chicken, mouse-chimpanzee, mouse-dog, mouse-cow, and mouse-chicken. Click here for file

Additional file 3 **HomoloGene data for human-rat orthologous gene pairs**. This pdf file contains HomoloGene data for human-rat orthologous gene pairs. Click here for file
