# Supplementary material for: Divergence of imprinted genes during mammalian evolution
Source: BMC Evol Biol. 2010 Apr 29;10:116. doi: 10.1186/1471-2148-10-116 (PMC2875234; doi:10.1186/1471-2148-10-116)
Supplement: Additional file 2 — HomoloGene data for additional orthologous gene pairs. This pdf file contains HomoloGene data for orthologous gene pairs of human-chimpanzee, human-cow, human-dog, human-chicken, mouse-chimpanzee, mouse-dog, mouse-cow, and mouse-chicken. [file 1471-2148-10-116-S2.PDF]

HomoloGene data for additional orthologous gene pairs

pairs	group	genes[a]	protein identity (%)	cDNA identity (%)	Ka/Ks	Ks
human-chimpanzee	imprinted	45 / 43	97.5±5.4	98.0±4.2	0.284±0.331	0.037±0.068
	mat	22	97.1±6.3	97.6±4.8	0.222±0.252	0.046±0.078*
	pat	23	98.0±4.4	98.4±3.6	0.350±0.394	0.028±0.058*
	genome	15,848 / 14,661	98.4±3.5	98.7±2.7	0.320±0.412	0.028±0.090
human-cow	imprinted	40	86.2±10.6	86.3±6.6	0.165±0.130	0.484±0.253
	mat	22	86.0±10.0	86.3±6.1	0.183±0.144	0.454±0.193
	pat	18	86.5±11.6	86.4±7.3	0.142±0.109	0.521±0.313
	genome	14,647 / 14,635	87.9±10.4	87.7±5.8	0.152±0.132	0.439±0.187
human-dog	imprinted	48	90.6±7.9	89.4±4.9	0.126±0.102	0.384±0.128
	mat	25	88.9±9.3	88.2±5.7	0.141±0.120	0.411±0.134
	pat	23	92.4±5.9	90.6±3.5*	0.109±0.079	0.354±0.116*
	genome	14,933 / 14,925	89.2±9.4	88.6±5.4	0.142±0.123	0.408±0.172
human-chicken	imprinted	40 / 31	74.8±12.5	72.9±7.9	0.125±0.096	1.470±0.500
	mat	24 / 18	74.4±12.5	72.8±7.8	0.134±0.095	1.425±0.530
	pat	16 / 13	75.4±12.9	73.0±8.2	0.112±0.100	1.532±0.468
	genome	11,201 / 9,614	75.2±15.3	73.4±8.8	0.116±0.098	1.651±0.740
mouse-chimpanzee	imprinted	42	83.9±11.5	83.3±6.8	0.145±0.112	0.667±0.254
	mat	22	83.8±9.8	83.0±6.0	0.152±0.113	0.667±0.221
	pat	20	84.0±13.4	83.6±7.8	0.137±0.114	0.668±0.291
	genome	13,988 / 13,962	85.0±11.9	84.1±6.6	0.133±0.113	0.650±0.234
mouse-cow	imprinted	38	80.6±12.2**	80.6±7.5**	0.154±0.118*	0.820±0.383
	mat	22	80.4±11.5*	80.4±7.2*	0.163±0.129	0.792±0.289
	pat	16	80.9±13.5	81.0±8.1	0.143±0.105	0.858±0.492
	genome	14,682 / 14,648	84.2±12.3	82.8±6.9	0.127±0.106	0.740±0.254
mouse-dog	imprinted	46	83.9±10.5	82.7±6.6	0.128±0.082	0.735±0.260
	mat	25	82.7±11.5	81.7±7.2	0.131±0.086	0.759±0.228
	pat	21	85.3±9.2	83.7±5.7	0.123±0.079	0.707±0.298
	genome	14,761 / 14,735	85.5±11.5	83.6±6.5	0.118±0.098	0.715±0.247
mouse-chicken	imprinted	38 / 27	72.5±11.8	71.0±7.2	0.123±0.084	1.739±0.618
	mat	25 / 19	72.8±12.4	71.3±7.5	0.122±0.089	1.770±0.642
	pat	13 / 8	71.9±11.0	70.4±6.8	0.125±0.076	1.668±0.593
	genome	11,083 / 8,599	74.1±15.6	72.0±8.9	0.106±0.088	1.875±0.773

[a]The second number refers to sequences available in the HomoloGene database for Ks and Ka/Ks analyses. For 100% identical sequences as well as for those with Ks reported as -1, Ka/Ks is not defined. Due to saturation, Ks becomes very high for human-chicken sequences.

Average and standard deviation are given. p values (Wilcoxon test) refer to comparison of genome-wide data with the respective imprinted group: * $p<0.1$, ** $p<0.05$

mat: maternally expressed; pat: paternally expressed
