# Supplementary material for: Divergence of imprinted genes during mammalian evolution
Source: BMC Evol Biol. 2010 Apr 29;10:116. doi: 10.1186/1471-2148-10-116 (PMC2875234; doi:10.1186/1471-2148-10-116)
Supplement: Additional file 3 — HomoloGene data for human-rat orthologous gene pairs. This pdf file contains HomoloGene data for human-rat orthologous gene pairs. [file 1471-2148-10-116-S3.PDF]

HomoloGene data for human-rat orthologous gene pairs

group	genes[a]	protein identity (%)	cDNA identity (%)	Ka/Ks	Ks
**imprinted**	47	83.1±11.6	83.1±6.4	0.152±0.113	0.672±0.284
**maternally expressed**	25	82.2±10.5	82.3±5.7	0.161±0.113	0.676±0.181
**paternally expressed**	22	84.3±12.8	84.0±7.2	0.141±0.113	0.667±0.374
**genome**	15,147/ 15,133	85.5±11.7 (p=0.086/p=0.054)	84.2±6.4 (p=0.105/p=0.046)	0.128±0.109 (p=0.086/p=0.076)	0.653±0.227 (p=0.775/p=0.269)

[a]The second number refers to sequences available in the HomoloGene database for Ks and Ka/Ks analyses. Average and standard deviation are given. The p values (Wilcoxon test) on the left refer to all imprinted genes, those on the right to genes with maternal expression in human. Paternally expressed genes do not differ from the genome (p>0.4).
